# Expanding the molecular toolkit for the homoacetogen *Clostridium ljungdahlii*

**DOI:** 10.1038/srep31518

**Published:** 2016-08-16

**Authors:** Bastian Molitor, Kristina Kirchner, Alexander W. Henrich, Simone Schmitz, Miriam A. Rosenbaum

**Affiliations:** 1 Institute of Applied Microbiology, Aachen Biology and Biotechnology, RWTH Aachen University, Germany

## Abstract

Increasing interest in homoacetogenic bacteria for the production of biochemicals and biofuels requisites the development of new genetic tools for these atypical production organisms. An attractive host for the conversion of synthesis gas or electricity into multi-carbon compounds is *Clostridium ljungdahlii*. So far only limited achievements in modifying this organism towards the production of industrially relevant compounds have been made. Therefore, there is still a strong need for developing new and optimizing existing genetic tools to efficiently access its metabolism. Here, we report on the development of a stable and reproducible transformation protocol that is applicable to *C. ljungdahlii* and several other clostridial species. Further, we demonstrate the functionality of a temperature-sensitive origin of replication in combination with a fluorescence marker system as important tools for future genetic engineering of this host for microbial bioproduction.

The genus *Clostridium* is a diverse group of Gram-positive, obligate anaerobic, rod-shaped and endospore-forming bacteria^1^,^2^. Within this group are both pathogenic (e.g. *Clostridium botulinum, Clostridium tetani, Clostridium perfringens*) and industrially relevant species (e.g. *Clostridium acetobutylicum, Clostridium kluyveri, Clostridium beijerinckii*)^3^,^4^,^5^,^6^. In recent years, homoacetogenic Clostridia (e.g. *Clostridium ljungdahlii, Clostridium autoethanogenum*) have attracted increasing attention^7^ due to their ability to fix CO_2_ via the Wood-Ljungdahl pathway^8^. This feature makes them attractive production hosts: 1) for the fermentation of synthesis gas, an energy rich gas composed of mainly CO_2_, CO and H_2_^7^,^9^,^10^,^11^,^12^,^13^,^14^; and 2) for a process known as microbial electrosynthesis in which CO_2_ is biologically reduced with electrons from a cathode^15^,^16^,^17^. Despite their interesting properties, molecular tools to investigate and engineer these interesting new biotechnological hosts are scarce, as was recently highlighted by Nybo *et al*.^18^. Several genetic methods for different acetogenic clostridial species have been developed^10^,^19^,^20^,^21^,^22^, but the transition of established methods from well-studied Clostridia, such as *Clostridium acetobutylicum*, for the application in homoacetogens is often challenging. The first genetic engineering progress in *C. ljungdahlii* was made for the heterologous production of butanol^23^, butyrate^24^, and acetone^25^. The genetic tools already developed for *C. ljungdahlii* include the development of a lactose-inducible promoter system^25^ and methods for the genomic integration of foreign DNA to either knock out genes through the integration of an antibiotic cassette or to integrate heterologous metabolic pathways^23^,^24^,^26^. Also, the utilization of a CRISPR/Cas9 system for gene-editing in *C. ljungdahlii* has been demonstrated recently^27^. However, many of the current techniques – starting with the transformation of *C. ljungdahlii* with foreign DNA – are still very inefficient^26^ and not very robust. There is a strong need for optimizing existing and further developing new genetic tools.

Here, we report on the development of a robust, stable and efficient transformation protocol for *C. ljungdahlii* and show its functionality for several other clostridial species. Further, we demonstrate the functionality of a temperature-sensitive origin of replication, combined with an anaerobic fluorescence marker, as important tools for future engineering of this versatile organism.

## Results

### An alternative transformation protocol for *C. ljungdahlii* is a versatile procedure that is effective for many Clostridia

The first transformation protocol for *C. ljungdahlii* was published in 2010^23^ (protocol 1; Table 1). The transformed plasmid pSOBP_ptb_ carried a Gram-positive origin of replication (ori^+^) derived from the plasmid pIMP1 (pIM13). Transformation efficiencies (E_t_) were not given in this publication. Based on protocol 1 an optimized procedure was published by Leang, *et al*.^26^ (protocol 2; Table 1). Depending on the used ori^+^, the authors reported E_t_ of up to 1.7 × 10^4^ transformants per μg of plasmid DNA for plasmid pCL2 (ori^+^: pIP404, Table 2). With the same transformation protocol, select examples of replicable plasmids from the modular pMTL80000 plasmid system (pMTL82151, ori^+^: pBP1; pMTL83151, ori^+^: pCB102) for *Clostridium* spec.^28^, yielded E_t_ of ~3 × 10^3^ transformants per μg of plasmid DNA. Protocol 2 was used in follow-up publications from the same group (without giving information about E_t_) to: 1) transform a suicide plasmid; the authors reported that one single colony grew after transformation and that a single-crossover genome integration event was observed (Table 2)^29^, 2) transform different plasmids with pIP404 as ori^+^ (Table 2)^25^, and 3) construct several strains carrying replicable plasmids and to construct several knock-out strains via either single cross-over homologous recombination or using the ClosTron system (Table 2)^24^. In the recent patent literature, a slightly modified protocol 2 was used to transform *Clostridium autoethanogenum*, a very closely related species to *C. ljungdahlii*^30^. E_t_ between 1.3 × 10^2^ and 1.3 × 10^3^ transformants per μg of plasmid DNA were reported in this patent application, depending on different methylation patterns^31^ (protocol 3; Table 1). To our knowledge no other transformation procedures for *C. ljungdahlii* or further use of the reported protocols are published so far.

Protocols 1 or 2 did not efficiently and reproducibly result in transformants in our hands. Therefore, we developed an alternative transformation procedure, which we here demonstrate to be applicable to different clostridial species. These species were not necessarily closely related to each other, showing the broad suitability of Gram^+^ replicons and the antibiotic resistance marker *catP* in different clostridial species. In Table 1 the differences of the published protocols and the final version of our new procedure (protocol 4) are summarized. The most important changes are the use of 10% glycerol instead of SMP buffer for washing steps and as electroporation solution as well as a prolonged outgrowth time of 24 to 48 h after electroporation. For this outgrowth, the undefined complex medium RCM was used instead of PETC medium. In our protocol, the plasmid DNA is methylated with the Φ3T I methyltransferase as described in Mermelstein and Papoutsakis^32^ using *Escherichia coli* strain DH5αMCR (K-strain, Dcm^+^ Dam^+^). In contrast, in all publications that utilize protocol 2^24^,^25^,^26^,^29^, the plasmid DNA is prepared from *E. coli* NEB Express (B-strain, Dcm^−^ Dam^+^) without external, site-specific methylation. We found robust and reproducible E_t_ values for *C. ljungdahlii* of 3.23 × 10^2^–3.23 × 10^3^ transformants per μg of plasmid DNA for plasmids with different ori^+^s (Table 2). There were no successful transformants with our new protocol when the plasmid DNA for transformation was prepared from *E. coli* NEB Express cells.

We also tested how robust the protocol is to small variations. We found that a starting OD_600_ ranging from 0.25 to 0.71 did not considerably change the E_t_. Other factors like an increased amount of plasmid DNA (3 μg instead of 2 μg) also had no considerable effects.

Transformation efficiencies for other tested clostridial species are given in Table 3. In these cases only plasmid pMTL82151 was used for comparison (except for *C. acetobutylicum*). E_t_ values were in the same range as those found for *C. ljungdahlii* (4 × 10^2^–9 × 10^2^), except for *C. perfringens* for which an E_t_ of 4.5 × 10^3^ was reached.

To choose the appropriate amount of antibiotics for the different Clostridia, we either used published concentrations^26^,^28^,^33^ or determined a rough minimal inhibitory concentration (MIC) in liquid cultures as described in Materials and Methods. Strain-specific PCR and a PCR targeting the introduced plasmid were always used to exclude false positive results after each transformation (Fig. S1).

### The anaerobic fluorescent protein system pGlow-CK^XN^Bs2/Pp1 is applicable in *C. ljungdahlii*

**Figure 1:**
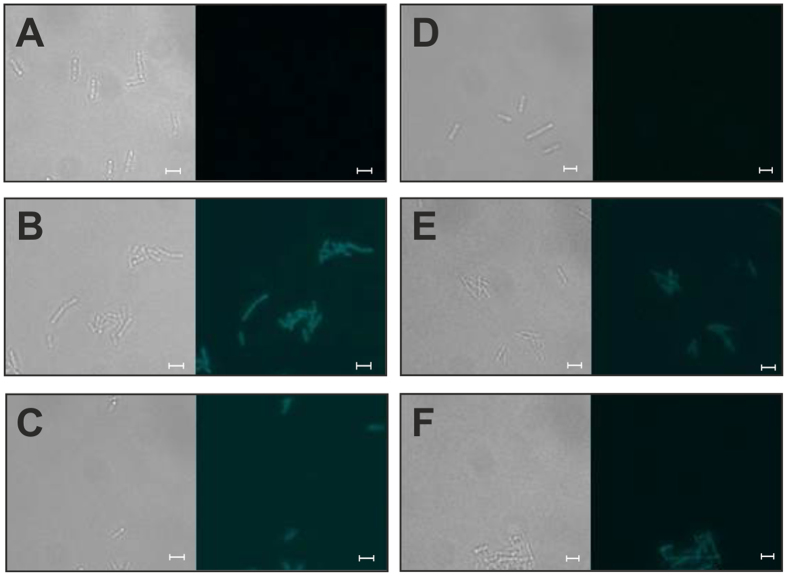
Fluorescence microscopic analyses of *C. ljungdahlii* wild type **(A)**, *C. ljungdahlii* (pGlow-CK^XN^Bs2) **(B)**, *C. ljungdahlii* (pGlow-CK^XN^Pp1) **(C)**, *C. acetobutylicum* wild type **(D)**, *C. acetobutylicum* (pGlow-CK^XN^Bs2) **(E)** and *C. acetobutylicum* (pGlow-CK^XN^Pp1) **(F)**. The left panels represent light microscopic images and the right panels show fluorescence microscopic images collected with a Leica DFC 365 FX fluorescence microscope equipped with a fluorescence cube 405 at excitation wavelength from 375–435 nm and emission wavelength of 445–495 nm. Scale bars, 3.23 μm.

The fluorescence marker system evoglow^®^ from Evocatal (Monheim, Germany) is known to be functional in *C. acetobutylicum* and is available as a clostridia-optimized commercial kit. The system is based on flavin mononucleotide (FMN)-based fluorescent proteins (FbFPs) that are – in contrast to GFP – functional under anaerobic conditions^34^. Here we applied this system in *C. ljungdahlii* and demonstrated the functionality for this organism. *C. acetobutylicum* was used as a control in these experiments. The intensity of the evoglow-CK^XN^-Bs2 and evoglow-CK^XN^-Pp1 proteins was not considerably different in fluorescence microscopic images (Fig. 1).

**Figure 2:**
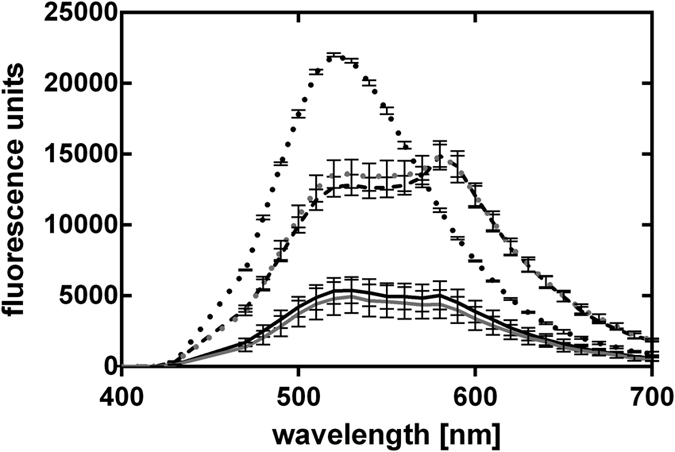
Fluorescence spectroscopic analyses of *C. acetobutylicum* (light grey) and *C. ljungdahlii* (black) wild type (solid lines) and strains carrying pGlow-CK^XN^Pp1 (dashed lines; not for *C. acetobutylicum)* or pGlow-CK^XN^Bs2 (dotted lines). Data represent the mean of n = 5. Error bars indicate standard deviation.

Fluorescence spectrometric measurements, however, showed slight differences in the fluorescence intensity, with the evoglow-CK^XN^-Bs2 variant giving higher fluorescence units than the evoglow-CK^XN^-Pp1 variant (Fig. 2).

### A temperature-sensitive origin of replication is a suitable tool to induce the loss of plasmids from cells

Plasmids with a conditionally suicidal origin of replication, i.e. the plasmids are only replicating under certain conditions, can be a useful tool for several applications. In these cases, it is important to maintain the functionality of the plasmid only for a given time. When the function should be shut off, the loss of the plasmid from the cells can be induced with one of several methods. For example, in *C. acetobutylicum* a system was established in which an inducible anti-sense RNA blocks the plasmid’s origin of replication leading to low segregational stability of the plasmid^35^. More commonly, temperature-sensitive origins of replication are described for several microorganisms and some of them are functional in a broad range of microbial hosts^36^,^37^,^38^,^39^. For example, the temperature-sensitive ori^+^ pWV01ts (pVE6002) derived from pWV01 from *Lactococcus lactis* subsp. *cremoris* was shown to be functional in *E. coli* and *Bacillus subtilis*^37^ and also was function as the origin in pSS60, used in *C. acetobutylicum*^40^. In this case, the temperature sensitivity results from four clustered mutations in the *repA* gene^37^ leading to stable maintenance at 30 °C and a non-permissive temperature of 37 °C.

**Figure 3:**
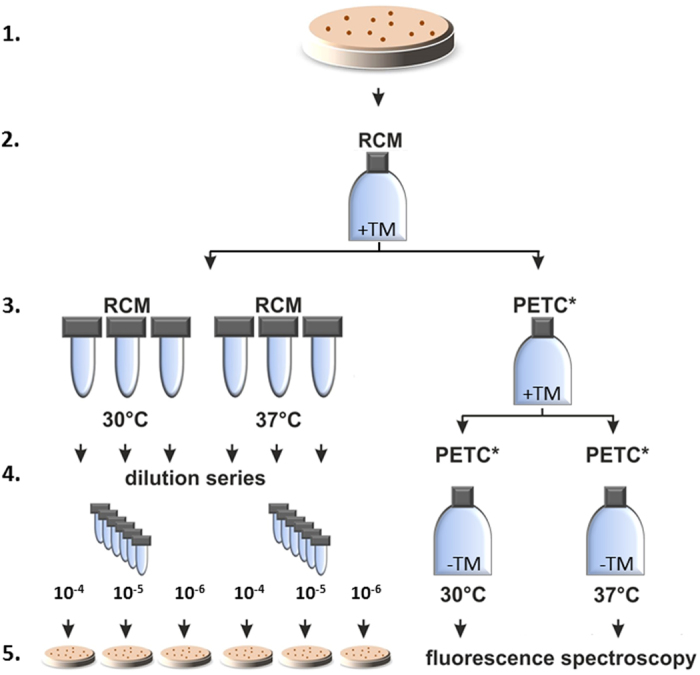
Schematic description of the experiments for induced plasmid loss. After a successful transformation of the plasmids (pMTLts, pMTLts_Bs2) **1.** one restreaking step was performed. **2.** A single colony was inoculated into RCM supplemented with thiamphenicol. **3. left** This preculture was used to inoculate six cultures in RCM without antibiotics. Three cultures were incubated at 30 °C and 37 °C, respectively. **4. left** Dilution series from the cultures from step 3 were performed from a similar starting OD and **5. left** 200 μl of the dilutions 10^−4^, 10^−5^ and 10^−6^ were plated onto selective RCM agar plates. Colonies were counted after incubation for 2–6 days at 30 °C. **3. right** For pMTLts_Bs2, a subculture of step 2 was inoculated in a serum bottle with PETC medium without yeast extract (PETC*). **4. right** This culture was then used to inoculate PETC* without antibiotics to incubate at 30 and 37 °C until sufficient growth was observed (~40 hours, minimum OD_600_ = 0.25); and **5. right** followed by subsequent fluorescence spectroscopic analyses. The wildtype was used throughout the experiments as a control for plating efficiencies without addition of antibiotics in any media.

**Figure 4:**
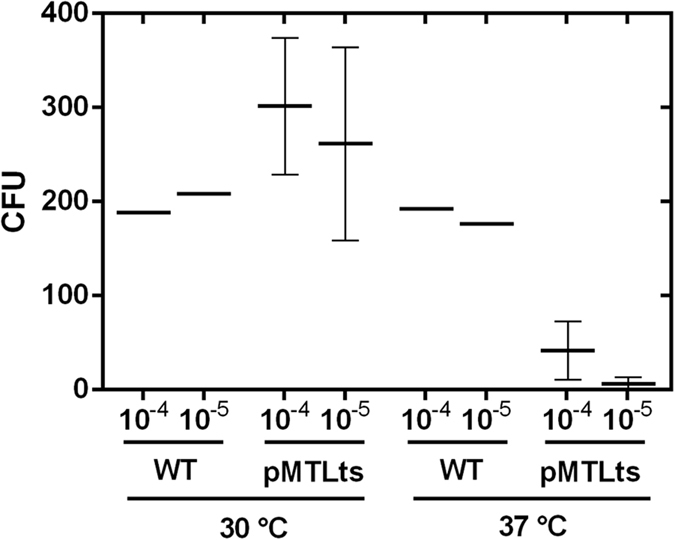
Stability of plasmid pMTLts in *C. ljungdahlii* without selective pressure under permissive (30 °C) and non-permissive (37 °C) temperatures. For *C. ljungdahlii* (pMTLts) thiamphenicol resistant cells were counted after plating 10^−4^ and 10^−5^ dilutions of the non-selectively grown cultures. For comparison of plating efficiencies *C. ljungdahlii* wildtype was plated in the same dilutions without thiamphenicol. WT (wildtype): n = 1; pMTLts (cells carrying plasmid pMTLts): n = 3. CFU = colony forming units.

We tested the pWV01ts origin of replication for functionality in *C. ljungdahlii*. Without a selection pressure (i.e. no antibiotics in the medium), the plasmid pMTLts was only maintained at the permissive temperature of 30 °C and was lost by dilution when cells were grown at 37 °C (Figs 3 and 4).

After one transfer and growth at 37 °C for 24 h, only 3–14% of the cells maintained the plasmid and were able to grow on thiamphenicol-containing RCM plates, compared to cultures grown at a permissive temperature of 30 °C (Fig. 4).

**Figure 5:**
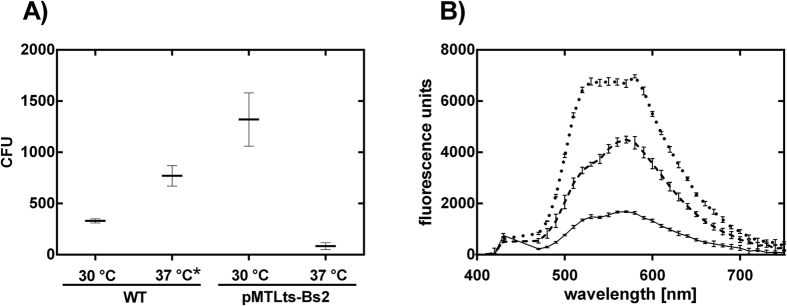
Stability of plasmid pMTLts_Bs2 in *C. ljungdahlii* without selective pressure under permissive (30 °C) and non-permissive (37 °C) temperatures according to the procedure from Fig. 3. (**A**) For *C. ljungdahlii* (pMTLts_Bs2) thiamphenicol resistant cells were counted after plating 10^−6^ dilutions of the non-selectively grown cultures. For comparison of plating efficiencies *C. ljungdahlii* wildtype was plated in the same dilutions without thiamphenicol. WT (wildtype): n = 3; pMTLts_Bs2: n = 3, except for 37 °C*: n = 2. (**B**) Confirmation of plasmid loss in *C. ljungdahlii* (pMTLts_Bs2) through the reduction of fluorescence intensity after incubation of liquid cultures in PETC* at permissive (30 °C) (dotted line) and non-permissive (37 °C) (dashed line) temperatures for ~40 hours. As a control the fluorescence intensity of the wildtype grown in the same medium at 37 °C is reported (straight line). CFU = colony forming units.

To further analyze the temperature-sensitive origin of replication and to demonstrate its usability in a functional plasmid, it was combined with the anaerobic fluorescence protein. For this purpose, the plasmid pMTLts_Bs2 was constructed, which carries the pWV01ts ori and the fluorescence marker *evoglow-Bs2-Cl* under the control of the constitutive thiolase promoter (P_thl_) from *C. acetobutylicum.* As expected, we observed the temperature-dependent decrease of fluorescence signal as the number of cells (CFU) carrying the plasmid pMTLts_Bs2 decreased (Fig. 5). The relationship was not linear, however, with ~40% reduction in fluorescence intensity vs. ~90% reduction of CFUs after incubation at 37 °C. This difference most likely results from the use of two different media. Quantitative colony counts utilized the preferred growth medium RCM during plasmid loss and plating, while fluorescence measurements of the Bs2 protein are only possible in defined PETC medium without the addition of yeast or beef extract. Hence, the growth rates were significantly different. Also, we do not have a quantitative measure for the specific growth phase or biomass correlated protein expression and fluorescence intensity of Bs2, which could be reflected in the fluorescence analysis.

### Compatibility with the pMTL80000 modular plasmid series

The plasmids pMTLts and pMTLts_Bs2 described here provide an additional ori^+^ (pWV01ts) and a fluorescent marker (evoglow-CK^XN^-Bs2) that are functional in *C. ljungdahlii* and *C. acetobutylicum*. According to the requirements of the modular pMTL80000 system^28^, the modularity/reversibility of the system via the four reserved restriction sites *Asc*I, *Fse*I, *Pme*I and *Sbf*I should be maintained for the integration of new modules. Further, important restriction sites in the multiple cloning site should remain exclusive and should therefore be avoided in the other modules. The fluorescent marker module that we integrated contained an additional *Sbf*I site. Thus, we performed a conservative mutation of this site (via QuikChange mutagenesis) to maintain the modularity criteria of the pMTL80000 system. With this modification, we suggest adding these modules to the nomenclature of the pMTL system and reserve numbers for the Gram^+^ replicon (e.g. pMTL86XXX) and the application-specific module (e.g. pMTL8XXX5).

## Discussion

We developed an electroporation protocol for *C. ljungdahlii* that is also applicable for several other clostridial species. Intensive application of the protocol in ongoing projects in our lab have confirmed its replicability. We achieved robust E_t_ values of 3.23 × 10^2^–3.23 × 10^3^ (Table 2) for plasmids with different ori^+^. To date, the highest reported E_t_ is 1.7 × 10^4^ for plasmid pCL2 with ori^+^ pIP404^26^, which was not tested with our protocol so far. Using this origin of replication might also lead to higher E_t_ with our protocol. Alternatively, the apparently lower E_t_ of our protocol might also be due to issues in the plating efficiency, which is highly variable for this obligate anaerobe.

One factor that seems to influence the efficiency greatly is the temperature of the molten RCM agar for the plating procedure. For plating the same volume from the same culture, we made the observation that fewer colonies arose when plates were poured at temperatures around 45 °C–50 °C, than when the agar was already cooled to approximately 40 °C. This makes it difficult to determine consistent E_t_ values and likely explains the high standard deviation in our measurements.

Further, in our experiments with *C. ljungdahlii,* initially not more than about 300–400 colonies grew, independent of the estimated number of plated cells (based on OD). Besides the agar temperature, specific effects of colony inclusion or the general fitness of cells might be influential. After a reorder of the strain from the German culture collection, significantly faster growth in liquid media and plating efficiency was observed (e.g., in the plasmid loss experiment with pMTLts_Bs2, Fig. 5). Laboratory degeneration of strains is observed frequently for solventogenic *Clostridium* sp.^41^,^42^, but this is the first report for acetogenic *Clostridium* strains. In our work, strain degradation probably reduced our plating efficiency and also E_t_ (E_t pMTLts_Bs2_ = 3.23 × 10^3^, Table 2) for the first part of this study.

Even despite these initial limitations in plating, our transformation protocol leads to a reproducible and sufficient amount of transformants when plating ~200 μl of a 5 ml liquid culture. For screening purposes, the number of plates can be increased to increase the number of clones.

We tested the anaerobic fluorescent marker system evoglow^®^ from Evocatal (Monheim, Germany) as a genetic tool for future functional engineering of *C. ljungdahlii*. Functionality was shown for *C. ljungdahlii* as well as for the control *C. acetobutylicum.* Different from the oxygen-dependent fluorescent proteins of the GFP family, this system is based on flavin-dependent reactions. The choice of the correct medium is therefore critical for the application of these fluorescence marker proteins. Medium containing high amounts of riboflavin (e.g., the common *C. ljungdahlii* media RCM and PETC) gave high background fluorescence, making it impossible to detect fluorescing cells. The utilization of PETC medium without yeast and beef extract (PETC*) overcame this problem. Growth of *C. ljungdahlii* in the mineral medium without these compounds with fructose as carbon source was slower but still sufficient. A low background fluorescence in these cultures is important since the fluorescence quantum yield (Q_F_) of the flavin-based fluorescent proteins naturally is lower than typical oxygen-dependent GFP-type fluorescent proteins^34^,^43^. Overall, we have confirmed that the anaerobic fluorescence marker system evoglow^®^ can be a versatile functional tool for *C. ljungdahlii*.

We further applied the fluorescent marker evoglow-CK^XN^-Bs2 to test the functionality of a third genetic tool – a temperature-sensitive origin of replication for controlled plasmid loss. For the construction of plasmid pMTLts from plasmid pMTL85141, only the Gram-positive origin of replication (ori^+^: pIM13) was exchanged against the pWV01ts ori; the high-copy number ColE1 Gram-negative origin of replication necessary for shuttle plasmid activity was maintained. Interestingly, during the cloning procedure in *E. coli,* it turned out that the temperature sensitive ori was dominant over the ColE1 ori. Therefore, temperature sensitivity was also observed in *E. coli*. In experiments with induced plasmid loss in *C. ljungdahlii* for pMTLts and pMTLts_Bs2 we could show a plasmid loss of ~90% through incubation at the non-permissive temperature of 37 °C for 24 h. For pMTLts_Bs2, we were also able to confirm this plasmid loss through the reduction of Bs2 fluorescence. The visible reduction in fluorescence intensity thereby was a good qualitative measure for plasmid loss, while the plate counts on RCM medium provide quantitative information. The importance of this temperature sensitive plasmid tool is mainly for applications in strain engineering, in which a temporary action of a plasmid-based element is desired, e.g., a mutagenic element or a restriction enzyme for genome editing. Once the desired action is performed, the plasmid can be easily removed from the cells. An application for bioproduction processes is less likely, since the permissive growth temperature of 30 °C results in significantly slower growth than the optimum 37 °C.

New genetic tools for *C. ljungdahlii* are an important prerequisite to study its physiology and for its targeted modification and application as an industrial production host. Our robust transformation protocol, the demonstration of the functionality of an anaerobic fluorescence marker system and a temperature-sensitive origin of replication broaden the spectrum of tools to study and modify *C. ljungdahlii.*

## Materials and Methods

Details on plasmid construction and oligonucleotides can be found in the supplemental information.

### Bacterial strains and growth conditions

Bacterial strains and plasmids used in this study are listed in Table 4. *E. coli* was routinely grown at 37 °C or 30 °C in LB broth or on LB agar. *E. coli* DH5αMCR was used for plasmid construction and propagation. Clostridia were grown anaerobically in modified reinforced clostridial medium (RCM), containing (per L): 10 g peptone, 10 g beef extract, 3 g yeast extract, 5 g D(−)-fructose, 5 g NaCl, 1 g soluble starch, 0.5 g L-cysteine-HCl, 3 g sodium acetate (pH 6.8) at 37 °C or 30 °C under strictly anaerobic conditions. For spectroscopic measurements of fluorescence, cultures were grown in PETC medium (ATCC medium 1754) without yeast and beef extract. All Clostridia media were extensively flushed with N_2_ (99.99999%) and residual oxygen was removed with L-cysteine-HCl as reducing agent. All manipulations of Clostridia were performed in an anaerobic glove box (Coylab, USA) under an atmosphere of 5% H_2_, 25% CO_2_ and 70% N_2_. Media were supplemented with antibiotics when necessary in the following concentrations: For *E. coli*, chloramphenicol (30 μg/ml); erythromycin (500 μg/ml); carbenicillin (ampicillin) (50 μg/ml); for *C. acetobutylicum* and *C. ljungdahlii*, thiamphenicol (5 μg/ml), clarythromycin (4 μg/ml), erythromycin (50 μg/ml); for *C. pasteurianum* and *C. perfringens*, thiamphenicol (10 μg/ml). To determine the appropriate amount of antibiotic for *C. pasteurianum* and *C. perfringens,* the cultures were inoculated to an OD_600_ of 0.05 to 0.1 into 5 ml RCM supplemented with increasing amounts of antibiotics. The antibiotic concentration where clear growth inhibition was observed was doubled and used for further experiments.

For growth of Clostridia on solid media a pour-plating method was used. In detail, 20 ml freshly prepared RCM-agar (1.5% agar), cooled down to 40 °C–50 °C, were mixed with 200–1000 μL of liquid bacterial culture and the appropriate antibiotics and poured into a petri dish. The plates were incubated at 37 °C or 30 °C until single colonies were visible (3–7 days).

### Methylation of plasmids

To prepare a plasmid for transformation into Clostridia, the desired plasmid was co-transformed into *E. coli* DH5αMCR together with either pAN1^32^ or pANA1 (depending on the resistance cassette of the target plasmid) for methylation with the Φ3T I methyl transferase^32^ at the inner cytosines of the sequences GGCC and GCNGC. The methylated plasmids were isolated from *E. coli* with the Qiaprep Spin Kit (Qiagen, Germany) and used to transform Clostridia. To test for a successful methylation, a digestion with the methylation-sensitive restriction endonuclease *Hae*III (NEB, Germany, cuts the motif GGCC after the second guanine only when the first cytosine is not methylated) was performed and checked via agarose gel electrophoresis.

### Transformation of Clostridia

All steps including centrifugation were performed in an anaerobic glove box (Coylab, USA) under a gas atmosphere of about 5% H_2_, 20% CO_2_, 75% N_2_. For a pre-culture, 50 ml RCM was inoculated to an OD_600_ of 0.05 to 0.1 and incubated at 37 °C overnight. For each transformation 9 ml of cell suspension (OD_600_ 0.2–0.8) was stepwise harvested in a 1.5 ml reaction tube by centrifugation (2000 × g, 1 min) and cell pellets were washed twice with 1.5 ml ice-cold 10% glycerol. Subsequently, the washed cells were resuspended in 200 μl of ice-cold 10% glycerol, 2 μg of methylated plasmid DNA was added and the mixture was directly transferred into a precooled electroporation cuvette with a gap-size of 0.2 cm (Labomedic, Germany). Electroporation was performed at the following conditions: 2.5 kV, 600 Ω, 25 μF. Directly after electroporation, the cells were transferred into 5 ml pre-warmed RCM and incubated for 24–48 h at 37 °C (or 30 °C for plasmids carrying pWV01ts origin of replication). After this non-selective outgrowth step, 200–1000 μl of culture were plated using the pour-plating method described above. The plates were incubated at 37 °C (or 30 °C) until colonies were obtained, which could take between 1–2 days for *C. acetobutylicum, C. perfringens, C. pasteurianum* and up to a week for *C. ljungdahlii*. The transformants were confirmed by colony PCR with plasmid-specific primers ColEI_for/ColEI_rev (Table S1) after one restreaking step or subcultivation in liquid RCM to avoid false positives due to residual plasmid DNA or untransformed wild type cells from the transformation procedure. Additionally, the integrity of the used strain was checked by colony PCR with strain-specific primers (fdhA_for/fdhA_rev, *C. ljungdahlii;* ctfAB_for/ctfAB_rev, *C. acetobutylicum;* nifC_for/nifC_rev, *C. pasteurianum;* luxS_for/luxS_rev, *C. perfringens;* Table S1).

Transformation efficiencies E_t_ were calculated with the following equation:


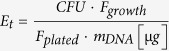


with 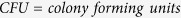, 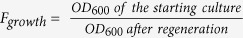, 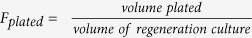, and 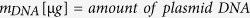.

### Fluorescence spectroscopic and microscopic analyses

*C. ljungdahlii* or *C. acetobutylicum* cells carrying pGlow-CK^XN^Pp1, pGlow-CK^XN^_Bs2^34^ or pMTLts_Bs2 were inoculated to an OD_600_ of 0.05 to 0.1 in RCM medium and incubated until exponential growth phase was reached at 37 °C or 30 °C with the addition of clarithromycin or thiamphenicol, respectively. These cultures were used to inoculate PETC medium without yeast and beef extract to an OD_600_ of 0.05 to 0.1 and incubated at the required temperature before fluorescence microscopic and spectroscopic analyses.

Fluorescence spectroscopic measurements in 200 μL volume at an OD_600_ of ~0.7 were performed in 96-well plates (Fluotrac 200 clear flat bottom black plates, Greiner Bio-One, Germany) in a plate reader (SynergyMX, BioTek, Germany), whereby the cultures were pelleted and diluted to adjust the OD. The emission spectra were recorded between 400 nm and 700 nm at an excitation wavelength of 450 nm.

Fluorescence microscopic pictures were captured with a fluorescence microscope (Leica DM6000B, Leica Microsystems, Germany) equipped with a fluorescence cube (LED405, Leica Microsystems, Germany) with an excitation wavelength filter of 375–435 nm and an emission wavelength filter of 445–495 nm and a monochrome digital CCD camera (Leica DFC 365 FX, Leica Microsystems, Germany).

### Induced plasmid loss experiments

A schematic description of this experiment is given in Fig. 3. In detail, *C. ljungdahlii* cells carrying pMTLts or pMTLts_Bs2 were inoculated in RCM with thiamphenicol to an OD_600_ of 0.05 to 0.1 and incubated for 24 h at 30 °C. 200 μl of the pre-culture was plated using the pour-plating method on selective agar. Single colonies were transferred into 5 ml liquid RCM containing thiamphenicol and the culture was incubated at 30 °C until exponential growth phase was reached. The culture was used to inoculate six new culture tubes with 5 ml RCM without antibiotics and three tubes each were incubated at 30 °C and 37 °C, respectively. For the determination of CFUs, a serial dilution of these cultures was prepared and 100 μl of the 10^−4^, 10^−5^, and 10^−6^ dilution was plated with the pour-plating method with the addition of thiamphenicol (three plates per dilution). Thereby, the starting OD for the serial dilution was comparable for the different culturing temperatures. The plates were incubated at 30 °C until colonies were obtained and counted.

To evaluate the temperature induced loss of the plasmid after cultivation at 30 °C and 37 °C in RCM, cultures carrying pMTLts_Bs2 were also transferred into PETC medium without yeast and beef extract and grown to an OD_600_ of at least 0.25. Fluorescence spectrometric measurements were then performed as described above (Fig. 3 right).

## Additional Information

**How to cite this article**: Molitor, B. *et al*. Expanding the molecular toolkit for the homoacetogen *Clostridium ljungdahlii. Sci. Rep.* **6**, 31518; doi: 10.1038/srep31518 (2016).

## Supplementary Material

Supplementary Information

## Figures and Tables

**Table 1:** Comparison of published procedures and new transformation protocol for *C. ljungdahlii*.

	Köpke *et al*., 2010	Leang *et al*., 2012	Reeves, 2014	New protocol
protocol No.	1	2	3	4
**preparation of cells and plasmids**				
OD_600_ (harvesting)	0.3–0.7	0.2–0.3	0.3–0.7	0.2–0.7
wash-buffer	SMP^a^	SMP	SMP	glycerol (10%)
pH wash-buffer	7.4	6	6	6
centrifugation steps	inside the chamber	outside of chamber	outside of chamber	inside the chamber
resuspension-buffer	SMP	SMP with 10% DMSO	SMP with 15% DMSO	glycerol (10%)
pH of res.-buffer	7.4	7.4	6.1	6
cell density at transformation	80-fold^b^	1000-fold	100-fold	30-fold
freeze/thaw	no	yes	yes	no
plasmid-methylation	yes^32^	no	yes (Clostridium spec. Type I; Reeves^31^)	yes^32^
strain plasmid-prep	K strain (ER2275)	B strain (NEB express)	several^c^	K strain (DH5αMCR)
**electroporation process**				
preincubation with plasmid on ice	5 min.	no	no	1–2 min.
volume of cells [μL]	600	25	50	200
plasmid-amount [μg]	0.1–1.5	1–5	1	2–3
electric pulse	2.5 kV, 600 Ω, 25 μF	0.625 kV, 600 Ω, 25 μF	1.5–2.5 kV, 600 Ω, 25 μF	2.5 kV, 600 Ω, 25 μF
electroporation cuvettes gap [cm]	0.4	0.1	0.2	0.2
cultivation after transformation	5 ml PETC, 37 °C	10 ml PETC, 37 °C	4 ml fermentation medium, 37 °C	5 ml RCM, 37 °C
outgrowth-cultivation time after transformation	until growth occurs	9–12 h	the next day	24–48 h
plating	liquid culture on solid agar	liquid culture mix with molten agar	liquid culture on solid agar	liquid culture mix with molten agar
antibiotics [μg/mL]	thiamphenicol: 20	thiamphenicol: 5	NI^d^	thiamphanicol: 5
	clarithromycin: 5	clarithromycin: 4	NI	clarithromycin: 4
organisms transformed with procedure	*C. ljungdahlii*	*C. ljungdahlii*	*C. autoethanogenum*	*C. ljungdahlii*
				*C. acetobutylicum*
				*C. perfringens*
				*C. pasteurianum*

^a^SMP = 270 mM sucrose, 1 mM MgCl_2_, 7 mM phosphate buffer.

^b^Calculated from the cell density in the beginning.

^c^DH10B; BL21; GM2163; DH5α; ER2275.

^d^NI = not indicated.

**Table 2:** Transformation efficiencies for *C. ljungdahlii* with different plasmids.

Plasmid	Origin of replication (Clostridium)	E_t_^a^(transformants/μg DNA), mean ± SD (n)	Reference	Utilized protocol^b^
pSOBP_ptb	pIMP1	NI^c^	Köpke, *et al*.^23^	1
pCL1	pIM13/pIMP1	1.1 ± 0.1 (3)	Leang, *et al*.^26^	2
pQexp	pAMβ1	14.9 ± 4.9 (6)	Leang, *et al*.^26^	2
pJIR750ai	pIP404	ND^d^	Leang, *et al*.^26^	2
pCL2	pIP404	(1.7 ± 0.6) × 10^4^ (5)	Leang, *et al*.^26^	2
pMTL82151	pBP1	(3.8 ± 0.2) × 10^3^ (3)	Leang, *et al*.^26^	2
pMTL83151	pCB102	(3.1 ± 1.8) × 10^3^ (3)	Leang, *et al*.^26^	2
pCR2.1rnfD::Cla^r	suicide^e^	1 colony	Tremblay, *et al*.^29^	2
pAH2	pIP404	NI^c^	Banerjee, *et al*.^25^	2
pKRAH1	pIP404	NI^c^	Banerjee, *et al*.^25^	2
pB1/pB2/pB3	pIP404	NI^c^	Banerjee, *et al*.^25^	2
pJe-p	pIP404	NI^c^	Ueki, *et al*.^24^	2
pM6-p	pBP1	NI^c^	Ueki, *et al*.^24^	2
pACR1/pACR1 (m)	pIMP1^f^	1.18 × 10^2^–1.333 × 10^3^	Reeves^31^	3
pMTL82151	pBP1	(5.55 ± 2.34) × 10^2^ (4)^g^	this work	4
pMTL83151	pCB102	(3.23 ± 2.02) × 10^2^ (5)^g^	this work	4
pMTLts	pWV01ts	(4.29 ± 2.97) × 10^2^ (9)^g^	this work	4
pGlow-CK^XN^Pp1	pIM13/pIMP1	(9.35 ± 8.45) × 10^2^ (5)^g^	this work	4
pGlow-CK^XN^Bs2	pIM13/pIMP1	(6.89 ± 3.58) × 10^2^ (8)^g^	this work	4
pMTLts_Bs2	pWV01ts	(3.23 ± 0.74) × 10^3^ (6)^g^	this work	4

^a^E_t_ = transformation efficiency.

^b^Compare Table 1.

^c^NI = not indicated.

^d^ND = not detected.

^e^No origin of replication.

^f^Efficiencies given for transformation of *C. autoethanogenum.*

^g^Corrected for growth by OD (for details, see *Materials and Methods* section).

**Table 3:** Transformation efficiencies for different clostridial species with plasmid pMTL82151 (*catP*, pBP1) or pGlow-CK^XN^Pp1, pGlow- CK^XN^Bs2 (*ermB*, pIM13).

Organism	Plasmid	E_t_ a(transformants/μg DNA), mean ± SD (n)
*C. acetobutylicum*	pMTL82151	(4.61 ± 4.3) × 10^2^ (6)
	pGlow-CK^XN^Pp1	(9.03 ± 3.38) × 10^2^ (3)
	pGlow-CK^XN^Bs2	(9.09 ± 5.07) × 10^2^ (3)
*C. pasteurianum*	pMTL82151	(4.03 ± 3.3) × 10^2^ (6)
*C. perfringens*	pMTL82151	(4.61 ± 1.08) × 10^3^ (6)

^a^E_t_ = transformation efficiency.

**Table 4:** Bacterial strains and plasmids used in this work.

Strain or plasmid	Relevant characteristics	Source
strains		
*Clostridium ljungdahlii* DSM 13528	wildtype	DSMZ
*C. ljungdahlii* (pGlow-CK^XN^Pp1)	carrying plasmid pGlow-CK^XN^Pp1	this work
*C. ljungdahlii* (pGlow-CK^XN^Bs2)	carrying plasmid pGlow-CK^XN^Bs2	this work
*C. ljungdahlii* (pMTLts)	carrying plasmid pMTLts	this work
*C. ljungdahlii* (pMTLts-Bs2)	carrying plasmid pMTLts-Bs2	this work
*Clostridium acetobutylicum* DSM 792	wildtype	DSMZ
*C. acetobutylicum* (pGlow-CK^XN^Pp1)	carrying plasmid pGlow-CK^XN^Pp1	this work
*C. acetobutylicum* (pGlow-CK^XN^Bs2)	carrying plasmid pGlow-CK^XN^Bs2	this work
*Clostridium perfringens* DSM 756	wildtype	DSMZ
*Clostridium pasteurianum* DSM 525	wildtype	DSMZ
*E. coli* DH5αMCR	*F- endAI supE44 thi-J A- recAl gyrA96 relAI deoR A(lacZYA-argF)U169 08OdlacZAM15 mcrA A(mrr hsdRMS mcrBC)*	44
plasmids		
pGlow-CK^XN^Pp1	Gram^+^: pIM13, *ermB*; Gram^−^: ColE1, AmpR; application: *evoglow-Pp1-Cl*	Evocatal, Germany
pGlow-CK^XN^Bs2	Gram^+^: pIM13, *ermB*; Gram^−^: ColE1, AmpR; application: *evoglow-Bs2-Cl*	Evocatal, Germany
pMTL82151	Gram^+^: pBP1, *catP*; Gram^−^: ColE1, *catP*; application: MCS	28
pMTL83151	Gram^+^: pCB102; *catP*; Gram^−^: ColE1, *catP*; application: MCS	28
pSS60	Gram^+^: *ermB*, pWV01ts; Gram^−^: ColE1, AmpR	gift from Peter Dürre, Ulm, Germany
pMTL85141	Gram^+^: pIM13, *catP*; Gram^−^: ColE1, *catP*; application: MCS	28
pMTLts	Gram^+^: pWV01ts, *catP*; Gram^−^: ColE1, *catP*; application: MCS	this study
pMTLts-Bs2	Gram^+^: pWV01ts, *catP*; Gram^−^: ColE1, *catP*; application: *evoglow-Bs2-Cl*	this study
pAN1	Gram^−^: p15A, *catP*; application: Φ*3t*I methyltransferase	32
pJET_AmpR	subcloned AmpR cassette from pUC18	this study
pANA1	Gram^−^: p15A, AmpR; application: Φ*3t*I methyltransferase	this study
