# Supplementary material for: Expanding the molecular toolkit for the homoacetogen Clostridium ljungdahlii
Source: Sci Rep. 2016 Aug 16;6:31518. doi: 10.1038/srep31518 (PMC4985741; doi:10.1038/srep31518)
Supplement: Supplementary Information [file srep31518-s1.pdf]

Supplemental information for:

Expanding the molecular toolkit for the homoacetogen *Clostridium ljungdahlii*

B. Molitor, K. Kirchner, A.W. Henrich, S. Schmitz, M.A. Rosenbaum

This supplementary information contains additional information on plasmid construction and verification.

Plasmid constructions

Oligonucleotides used in this work are listed in Table S1. The plasmid pMTLts was constructed via Phosphorothioated Ligase Independent Cloning (PLICing) [44]. Therefore, the temperature sensitive origin of replication pWV01ts was PCR amplified from pSS60 (a kind gift from Peter Dürre, Ulm, Germany) with phosphorothioated (PTO) primers (Eurofins MWG, Germany) pWVfor/ pWVre. The plasmid backbone from pMTL85141 [27] was PCR amplified with PTO primers pMTLfor/pMTLrev. The PLICing was performed as described before [44]. In detail, the used PTO primers introduced phosphorothioated, complementary overhangs as indicated in Table S1. The PCR products were treated with iodine solution (100 mM in 99 % ethanol) at 70 °C

for 5 min, mixed in a 1:1 ratio, incubated for 5 min at room temperature and directly transformed into competent *E. coli* DH5αMCR.

The plasmid pMTLts-Bs2 was constructed using Gibson Assembly cloning (NEB, USA). The vector backbone pMTLts was digested with *Eco*RI (NEB, USA), the evoglow-Bs2-Cl gene was PCR amplified using primers thl_pGlow_Bs2_fwd/ thl_pGlow_Bs2_rev from pGlow-CK^{XN}-Bs2 (Evocatal, Germany) and both DNA fragments were used in a Gibson Assembly reaction to give pMTLts-Bs2 (Gibson Assembly kit, NEB, USA).

For the construction of pANA1 the ampicillin resistance cassette from pUC18 was PCR amplified with primers Amp_for*Bam*HI/ Amp_rev*Eco*RI and cloned into pJet2.1 giving pJet_AmpR. Subsequently, the *Bam*HI and *Eco*RI (NEB, USA) digested ampicillin resistance cassette from pJet_AmpR was subcloned into pAN1, digested with the same enzymes, giving pANA1.

Table S1. Oligonucleotides used in this study

	**name**	**sequence (5' -> 3')**
**1**	pWVfor	CACCACGTAGTTATTGGGAGGTC
**2**	pWVrev	CGTCTATGAGCCTCTGAGAG
**3**	pMTLfor	GGCTCATAGACGCTGCAATCGGATGCGATTAT
**4**	pMTLrev	AACTACGTGGTGTGCAATTGTATTGCTATTAATCGC
**5**	thl_pGlow_Bs2_fwd	catatgaccatgattacgAGCTCCTGCAGGTCGACTTTTTAAC
**6**	thl_pGlow_Bs2_rev	cgggtaccgagctcgTTATTCAAGAAGCTTTTCATATTCTTTTTGCTTTGTTATATCATTC
**7**	Amp_for*Bam*HI	*GGATCC*GGTCTGACGCTCAGTGGAAC
**8**	Amp_rev*Eco*RI	*GAATTC*GAAAGGGCCTCGTGATACGC
**9**	ColEI_for	CCACCACTTCAAGAACTCTG
**10**	ColEI_rev	GCGGTAATACGGTTATCCAC
**11**	fdhA_for	AGTGCAGCGTATTCGTAAGG
**12**	fdhA_rev	TAATGAGCCACGTCGTGTTG
**13**	ctfAB_for	ATAGGCAGCAACCCAGATAC
**14**	ctfAB_rev	TTTAGGACTAGCGCCCATTC
**17**	nifC_for	TCGGTATTCAGGTGGTTCTC
**18**	nifC_rev	AAAGTGGCTCCAAACTCTCC
**19**	luxS_for	ACAAAGGTTAAGGCACCATATGT
**20**	luxS_rev	ACCTGTTTTGCATGACTCTTAGCT

Underlined sequences represent phosphorothioated sequences; small letters represent overhangs for Gibson assembly; italic, underlined sequences represent restriction enzyme cutting sites

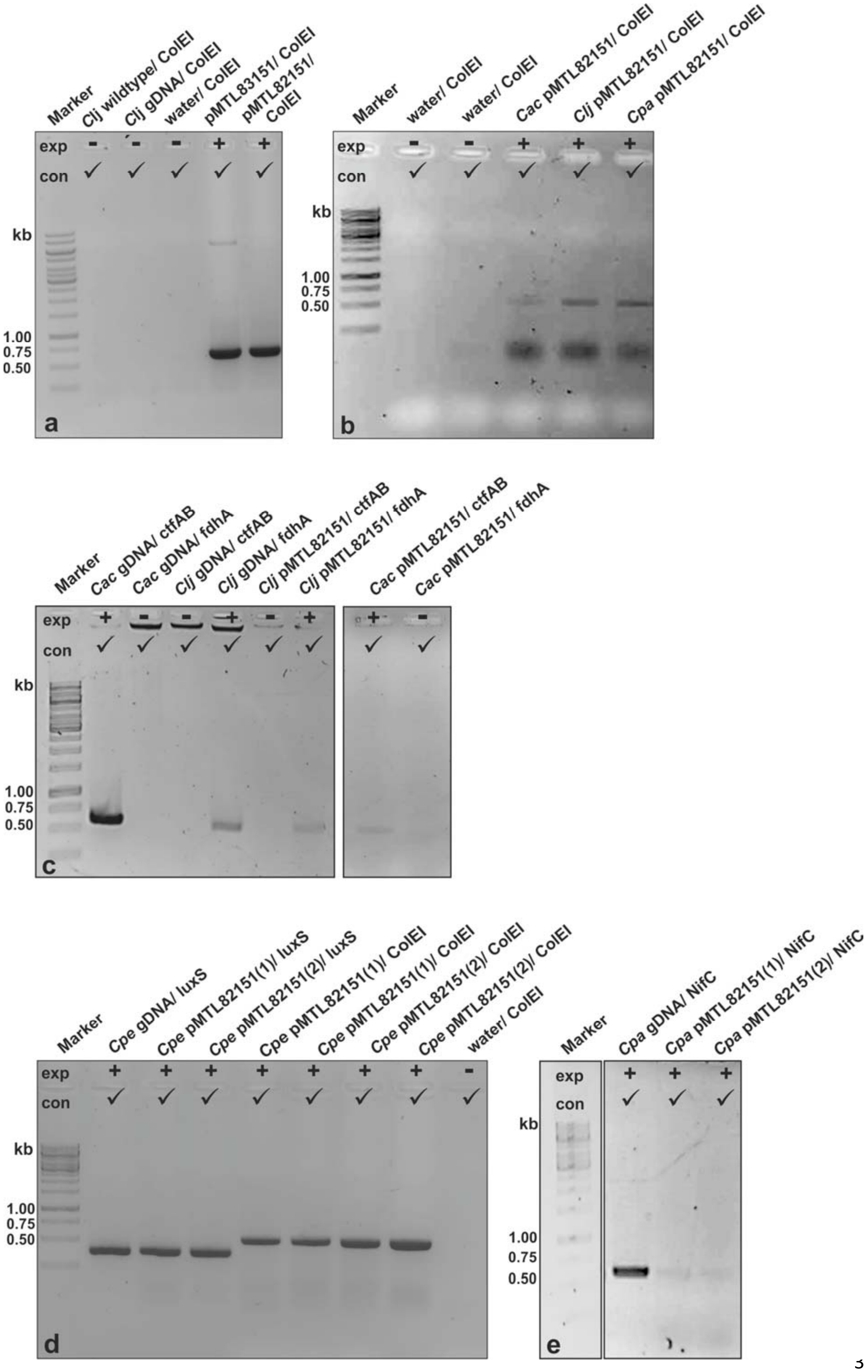

Marker
Clj wildtype/ ColEI
Clj gDNA/ ColEI
water/ ColEI
pMTL83151/ ColEI
pMTL82151/ ColEI
exp
con
kb
1.00
0.75
0.50
a
Marker
water/ ColEI
water/ ColEI
Cac pMTL82151/ ColEI
Clj pMTL82151/ ColEI
Cpa pMTL82151/ ColEI
exp
con
kb
1.00
0.75
0.50
b
Marker
Cac gDNA/ ctfAB
Cac gDNA/ fdhA
Clj gDNA/ ctfAB
Clj gDNA/ fdhA
Clj pMTL82151/ ctfAB
Clj pMTL82151/ fdhA
Cac pMTL82151/ ctfAB
Cac pMTL82151/ fdhA
exp
con
kb
1.00
0.75
0.50
c
Marker
Cpe gDNA/ luxS
Cpe pMTL82151(1)/ luxS
Cpe pMTL82151(2)/ luxS
Cpe pMTL82151(1)/ ColEI
Cpe pMTL82151(1)/ ColEI
Cpe pMTL82151(2)/ ColEI
Cpe pMTL82151(2)/ ColEI
water/ ColEI
exp
con
kb
1.00
0.75
0.50
d
Marker
Cpa gDNA/ NifC
Cpa pMTL82151(1)/ NifC
Cpa pMTL82151(2)/ NifC
exp
con
kb
1.00
0.75
0.50
e

Figure S1. Strain specific PCR and plasmid PCR to prove a successful transformation. Used primers are listed in Table S1. *Cac, C. acetobutylicum; Clj, C. ljungdahlii; Cpa, C. pasteurianum; Cpe, C. perfringens.* Marker, GeneRuler 1kb DNA Ladder (Fermentas): a = controls; b = plasmid confirmation strains *Cac*, *Clj*, *Cpa*; c = strain confirmation strains *Cac*, *Clj*; d = strain and plasmid confirmation *Cpe*; e = strain confirmation *Cpa*. (exp = expected result; con = confirmed).
